# Classification of microcalcification clusters in digital breast tomosynthesis using ensemble convolutional neural network

**DOI:** 10.1186/s12938-021-00908-1

**Published:** 2021-07-28

**Authors:** Bingbing Xiao, Haotian Sun, You Meng, Yunsong Peng, Xiaodong Yang, Shuangqing Chen, Zhuangzhi Yan, Jian Zheng

**Affiliations:** 1 Institute of Biomedical Engineering, School of Communication and Information Engineering, Shanghai University, Shanghai, China; 2 University of Science and Technology of China, Hefei, China; 3 Department of Medical Imaging, Suzhou Institute of Biomedical Engineering and Technology, Chinese Academy of Sciences, Suzhou, China; 4 Department of Breast Surgery, The Affiliated Suzhou Hospital of Nanjing Medical University, Suzhou, China; 5 Gusu School, Nanjing Medical University, Suzhou, China; 6 Department of Radiology, The Affiliated Suzhou Hospital of Nanjing Medical University, Suzhou, China

**Keywords:** Microcalcification cluster, Digital breast tomosynthesis, Convolution neural network, Ensemble learning, Classification

## Abstract

**Background:**

The classification of benign and malignant microcalcification clusters (MCs) is an important task for computer-aided diagnosis (CAD) of digital breast tomosynthesis (DBT) images. Influenced by imaging method, DBT has the characteristic of anisotropic resolution, in which the resolution of intra-slice and inter-slice is quite different. In addition, the sharpness of MCs in different slices of DBT is quite different, among which the clearest slice is called focus slice. These characteristics limit the performance of CAD algorithms based on standard 3D convolution neural network (CNN).

**Methods:**

To make full use of the characteristics of the DBT, we proposed a new ensemble CNN, which consists of the 2D ResNet34 and the anisotropic 3D ResNet to extract the 2D focus slice features and 3D contextual features of MCs, respectively. Moreover, the anisotropic 3D convolution is used to build 3D ResNet to avoid the influence of DBT anisotropy.

**Results:**

The proposed method was evaluated on 495 MCs in DBT images of 275 patients, which are collected from our collaborative hospital. The area under the curve (AUC) of receiver operating characteristic (ROC) and accuracy of classifying benign and malignant MCs using decision-level ensemble strategy were 0.8837 and 82.00%, which were significantly higher than the experimental results of 2D ResNet34 (AUC: 0.8264, ACC: 76.00%) and anisotropic 3D ResNet (AUC: 0.8455, ACC: 76.00%). Compared with the results of 3D features classification in the radiomics, the AUC of the deep learning method with decision-level ensemble strategy was improved by 0.0435, and the F1 score was improved from 79.37 to 85.71%. More importantly, the sensitivity increased from 78.13 to 84.38%, and the specificity increased from 66.67 to 77.78%, which effectively reduced the false positives of diagnosis

**Conclusion:**

The results fully prove that the ensemble CNN can effectively integrate 2D features and 3D features, improve the classification performance of benign and malignant MCs in DBT, and reduce the false positives.

## Background

Breast cancer has the highest morbidity and mortality among women's cancers [1], and early diagnosis and treatment can effectively improve the prognosis of breast cancer [2]. According to clinical statistics, 30% and 50% of breast cancers are accompanied by microcalcification clusters (MCs) [3]. As an important early manifestation of breast cancer, MCs is of great significance for early screening of breast cancer [4–6]. Correct classification of benign and malignant MCs by imaging examination is significant and can reduce unnecessary biopsy [7, 8].

Digital mammography (DM) and digital breast tomosynthesis (DBT) are now widely used to diagnose MCs. DM is considered to be the most reliable and effective method for breast cancer screen. However, there is overlap between the lesion and dense tissue in DM, which can easily lead to misdiagnosis of the MCs. DBT is an innovative imaging technique that can reconstruct 3D breast volume by acquiring low-dose mammogram projection views from a limited angle. It can overcome the effects of tissue overlap and improve the classification accuracy [9–11].

However, the following challenges still exist in the diagnosis of MCs in DBT. First, MCs is distributed in multiple slices of DBT, which is not conducive to the overall observation of the lesions. DBT images need to be scanned slice by slice, which brings a great workload to radiologists. Second, artifacts of microcalcification may be present due to reconstruction or potential movement of the patient, affecting the radiologist's diagnosis of MCs. Computer-aided diagnosis (CAD) system can assist radiologists in reading DBT images by automatically identify benign and malignant MCs, which can improve the diagnostic accuracy and efficiency for radiologists.

## Related works

To this end, various studies have been proposed for the CAD algorithm of MCs in DM and DBT. Fanizzi et al. [12] firstly performed multi-scale wavelet decomposition of the lesion area in the DM images, and extracted the texture features of each sub-image and its corresponding gray-level co-occurrence matrix, and the benign and malignant MCs were then classified using random forest (RF) [13] classifier. Considering the multi-scale connectivity relationship between microcalcifications, George et al. [14] extracted graph connectivity features at each scale to classify benign and malignant MCs in DM images. Zhang et al. [15] extracted radiomics features from the tomographic volume, projection image and focus slice of DBT, respectively, to classify whether the candidate was MCs or false positive sample. The results showed that the fusion of features of three data can effectively reduce false positives.

Among the most relevant tasks, Peng et al. [16] proposed a radiomics method to classify benign and malignant MCs. The method extracted 2D features from the maximum intensity projection image (MIP) and the focus slice, respectively, and 3D features were extracted from the tomographic volume. Finally, the comparative classification experiments of 3D features, 2D features and the combination of 3D features and 2D features were performed. The experimental results showed that the best result can be obtained when only 3D features before classification were used for classification, and the direct fusion of 2D features and 3D features in the radiomics method may not achieve better experimental results due to the feature redundancy.

All the above studies used the radiomics method to classify the MCs. However, radiomics method is based on artificially designed feature and a carefully selected classifier, so it has a limited generalizability. While deep learning (DL)-based method can extract features and classify them automatically, so DL-based method can easily generalize to new data.

At present, some DL methods have been proposed to classify MCs. Cai et al. [17] compared the classification performance of DL features and handcrafted features on benign and malignant MCs in DM. The experimental results showed that the classification result of DL features was better than handcrafted features. Considering the local characteristics of MCs and the surrounding tissue background, Wang et al. [18] proposed a context-sensitive deep neural network to reduce false positives, in which two CNNs were used to extract the features of MCs in DM at different scales. In the features-level ensemble, the feature vectors of two CNNs after global pooling were concatenated for the final classification. Samala et al. [19] designed a CNN to classify MCs detected in the prescreening stage. The method used the multiscale bilateral filtering regularized simultaneous algebraic reconstruction to classify the MIP of MCs in DBT, and classify whether the input image has MCs. MIP image is obtained by projecting DBT among *Z* axis, which is a compression of 3D information. So 3D image turn to MIP image may lose key information and lead misdiagnosis of the algorithm.

## Contributions

The main contributions of this study are as follows: (1) an ensemble CNN was proposed to classify benign and malignant MCs in DBT. The network can fuse the classification results of 2D intra-slice features and the 3D spatial features to improve the classification performance. To the best of our knowledge, this is the first ensemble CNN for classification of MCs in DBT. (2) The anisotropic 3D convolution was adopted to extract 3D spatial features, so as to avoid the influence of DBT anisotropic resolution. (3) The method was experimented on a clinical DBT dataset. We compared the classification performance of the proposed method with the independent 2D ResNet34 and anisotropic 3D ResNet, as well as with the representative radiomics method. Experimental results showed that the proposed ensemble CNN achieved the best performance.

## Results

### A. Performance evaluation measures

We drew the receiver operating characteristic (ROC) curve to visually compared the diagnostic performance between different models. In addition, we used the area under the ROC curve (AUC), accuracy (ACC), sensitivity (SEN), specificity (SPEC), precision, recall and *F*1 score to evaluate the performance of the models, where sensitivity describes the ability of the model to classify positive cases as positive. The lower the sensitivity, the more likely the model is to misdiagnose positive cases as negative cases. The specifically describes the ability of the model to classify negative cases as negative. The lower the specificity, the more likely the model lead missed diagnosis of malignant patients. *F*1 is the harmonic average of the two evaluation indexes, giving consideration to both precision and recall. The definitions of these criteria are as follows:

1 $${\text{ACC}} = \frac{{{\text{TP}} + {\text{TN}}}}{{{\text{TP}} + {\text{FP}} + {\text{TN}} + {\text{FN}}}},$$

2 $${\text{SEN}} = \frac{{{\text{TP}}}}{{{\text{TP}} + {\text{FN}}}},$$

3 $${\text{SPEC}} = \frac{{{\text{TN}}}}{{{\text{TN}} + {\text{FP}}}},$$

4 $${\text{Precision}} = \frac{{{\text{TP}}}}{{{\text{TP}} + {\text{FP}}}},$$

5 $${\text{Recall}} = \frac{{{\text{TP}}}}{{{\text{TP}} + {\text{FN}}}},$$

6 $$F1\;{\text{Score}} = \frac{{2 * {\text{Precision}} * {\text{Recall}}}}{{{\text{Precision}} + {\text{Recall}}}}.$$

The specific meanings of TP, TN, FP and FN are shown in Table 1.

**Table 1:** Two-class confusion matrix

True condition	Predicted condition	
	Positive	Negative
Positive	TP (true positive)	FN (false negative)
Negative	FP (false positive)	TN (true negative)

In the analysis of experimental results, we calculated the AUC of view level and lesion level, respectively. At the view level, the mediolateral oblique (MLO) and craniocaudal (CC) images of the same lesion were taken as independent lesion to calculate the criteria, respectively. At the lesion level, the MLO and CC images of the same lesion were seen as the same sample, and the final prediction scores were averaged.

### B. Comparison of 2D CNNs with different depths

**Fig. 1:**
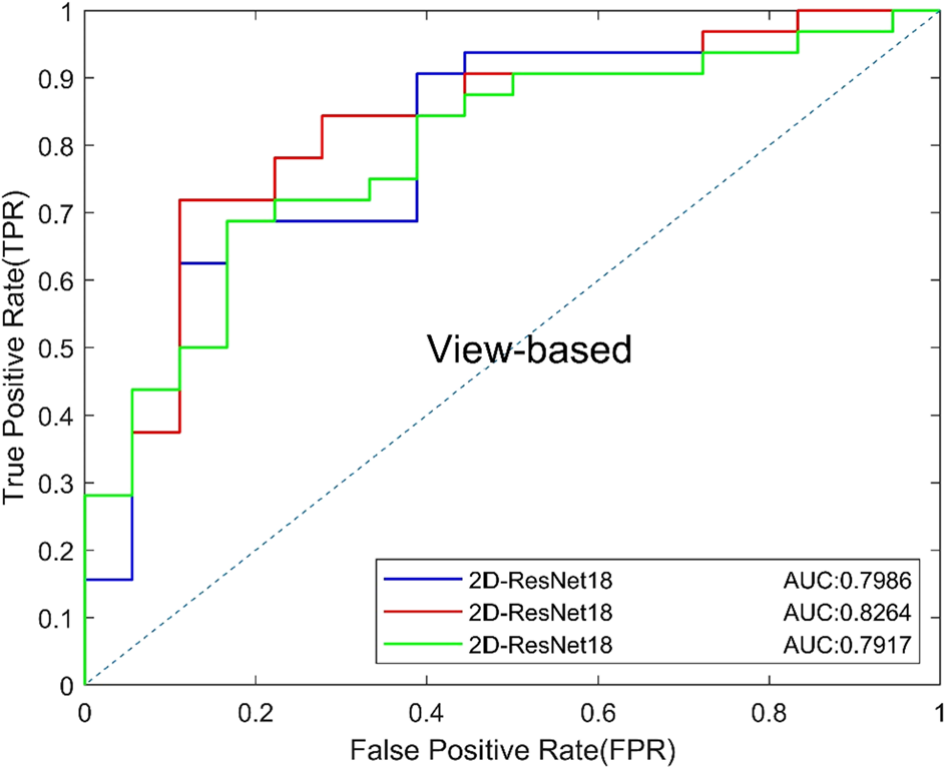
View-based ROC curves of 2D CNNs of different layers

**Fig. 2:**
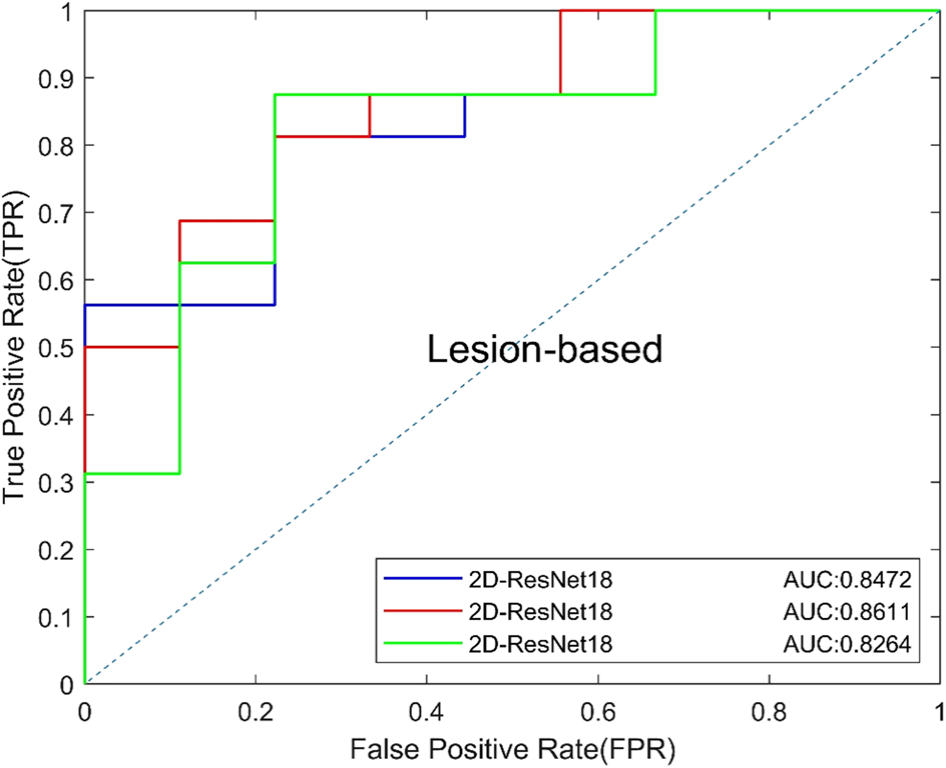
Lesion-based ROC curves of 2D CNNs of different layers

In the study of 2D CNN, the focus slice was selected as the input, and the image size was resized from 300 × 300 × 1 to 224 × 224 × 1. We firstly trained ResNet18, ResNet34 and ResNet50 to classify the focus slices and compared the performance between CNNs of different depths [20]. The results are shown in Table 2, and the ROC curves based on view and lesion are shown in Figs. 1 and 2. ResNet34 has the best performance on both view-based and lesion-based classification. The AUC of ResNet34 (0.8264) was higher than that of ResNet18 (0.7986) and ResNet50 (0.7917), and Resnet34 had the highest specificity. Results show that shallow networks cannot extract better features and have poor classification performance. Deep CNN can learn deep features of images, however, too many network layers can easily lead to overfitting, resulting in poor experimental results.

**Table 2:** Classification performance of 2D CNNs

Models	AUC	ACC (%)	SEN (%)	SPEC (%)	Precision (%)	Recall (%)	*F*1 (%)
2D-ResNet18	0.7986	74.00	81.25	61.11	78.79	81.25	80.00
2D-ResNet34	0.8264	76.00	78.13	72.22	83.33	78.13	80.65
2D-ResNet50	0.7917	72.00	75.00	66.67	80.00	75.00	77.42

### C. Comparison of different 3D CNNs

**Fig. 3:**
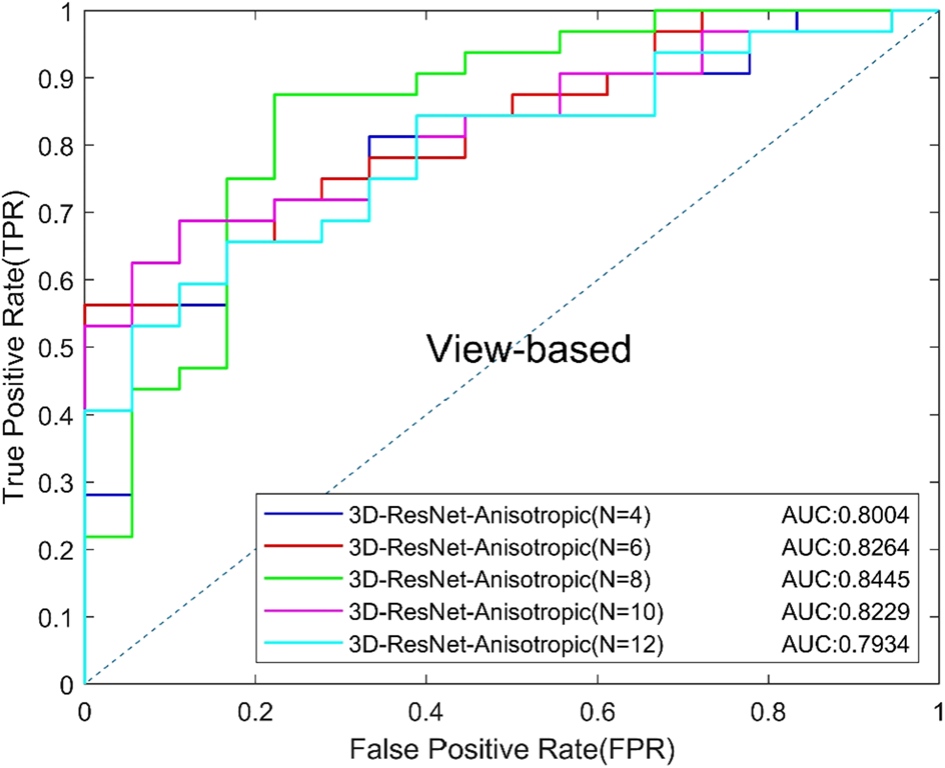
View-based ROC curves of different tomographic slices

**Fig. 4:**
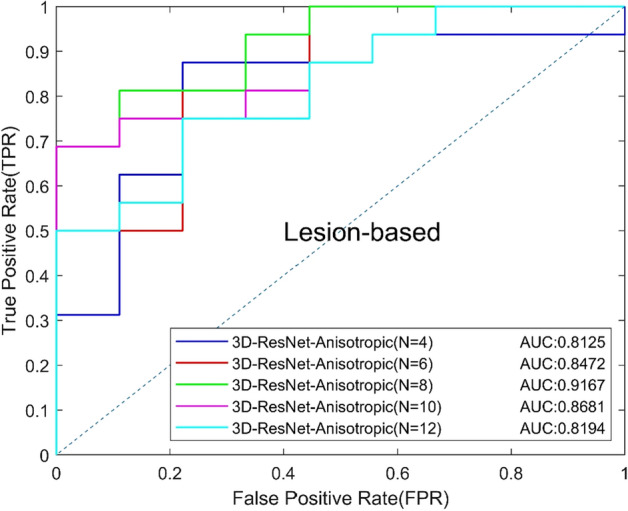
Lesion-based ROC curves of different tomographic slices

Different slice numbers of input can make a different impact on 3D classification results. We tested the effect of different input slices (*N* = 4, 6, 8, 10, 12) of the proposed anisotropic 3D ResNet. The results are shown in Table 3, and the ROC curves based on view and lesion are shown in Figs. 3 and 4. The experimental results show that the best result can be obtained when the number of slices is 8, and the model with 6 slices is better than that of 4 slices, which indicated that the more slices number, the more information will be provided, and the model can obtain better performance. However, the number of slices cannot be increased without limit, because the slice further away from the central slice, the more blurred appearance of MCs will be. Too many slices may provide invalid even confusing information for classification, which will increase the risk of over-fitting and the model is hard to generalize to new data. Increasing the number of input slices to 10 or 12 may incorporate some more blurred slices and reduce the performance of the model.

**Table 3:** Classification performance of different slices

Models	*N*	AUC	ACC (%)	SEN (%)	SPEC (%)	Precision (%)	Recall (%)	*F*1 (%)
3D-ResNet-Anisotropic	4	0.8004	76.00	84.38	61.11	79.41	84.38	81.82
	6	0.8264	72.00	71.88	72.22	82.14	71.88	76.67
	8	0.8455	76.00	75.00	77.78	85.71	75.00	80.00
	10	0.8229	74.00	84.38	55.56	77.14	84.38	80.60
	12	0.7934	70.00	75.00	61.11	77.42	75.00	76.19

**Fig. 5:**
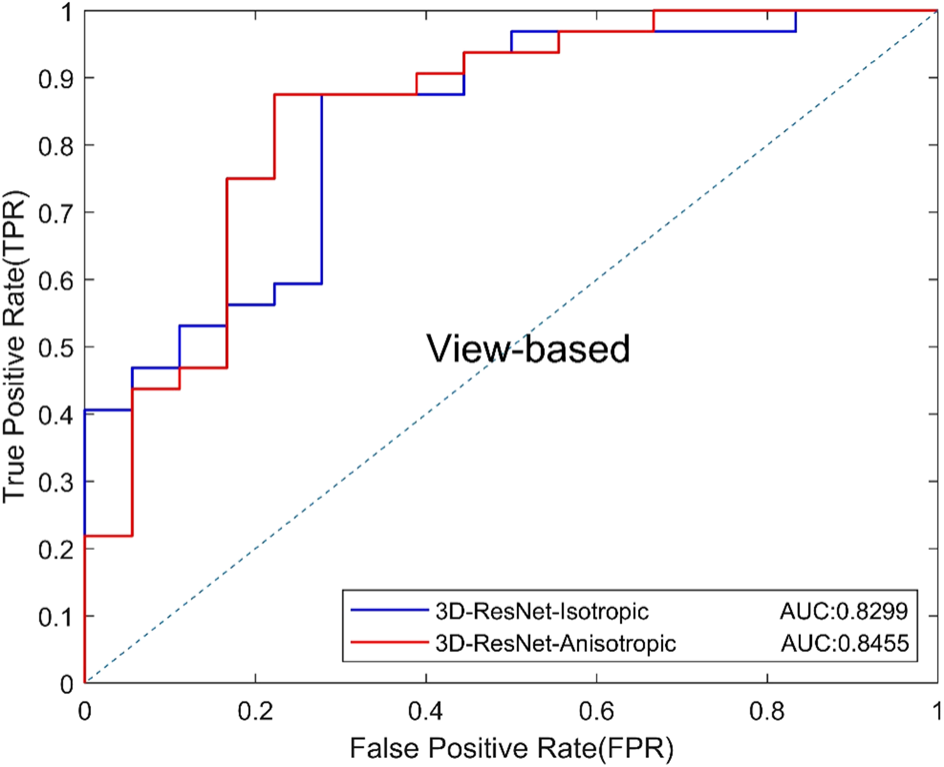
View-based ROC curves of different 3D CNNs

**Fig. 6:**
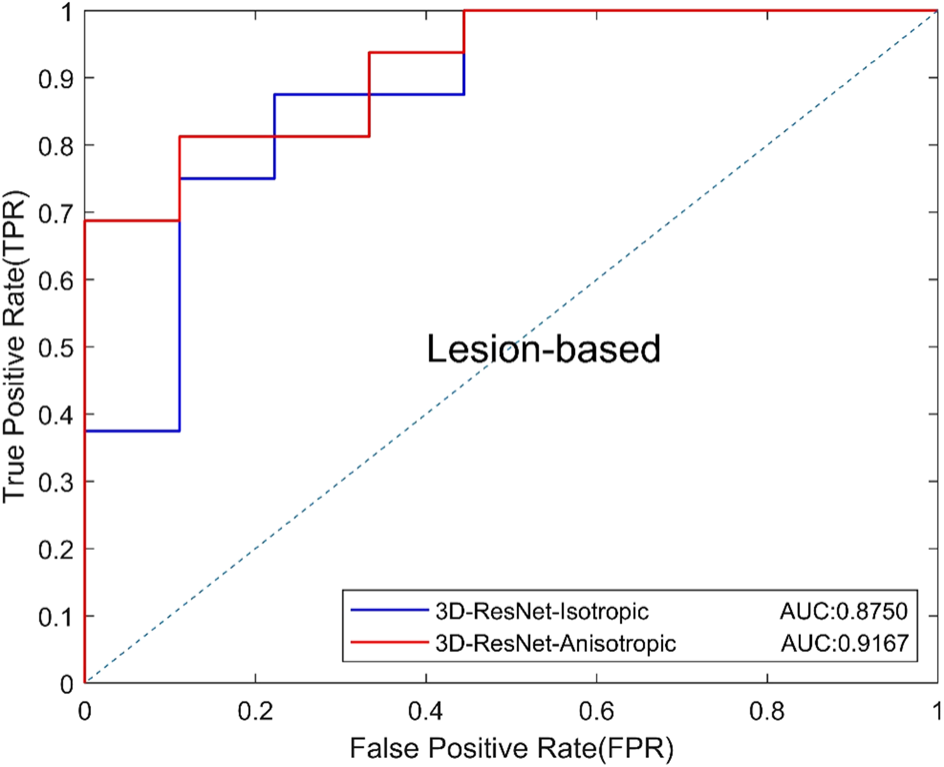
Lesion-based ROC curves of different 3D CNNs

Based on the above experiment, we used 8 slices as input to compare the proposed anisotropic 3D ResNet and the standard 3D ResNet. The results are shown in Table 4, and the ROC curves based on view and lesion are shown in Figs. 5 and 6. Results show that the anisotropic 3D ResNet had better AUC than standard 3D ResNet. In addition, the specificity increased from 61.11 to 77.78%, which means that anisotropic 3D ResNet can effectively reduce the false positives of classification of benign and malignant MCs.

**Table 4:** Classification performance of 3D CNNs

Models	AUC	ACC (%)	SEN (%)	SPEC (%)	Precision (%)	Recall (%)	*F*1 (%)
3D-ResNet-Isotropic	0.8299	78.00	87.50	61.11	80.00	87.50	83.58
3D-ResNet-Anisotropic	0.8455	76.00	75.00	77.78	85.71	75.00	80.00

### D. Comparison of different ensemble strategies

The above experiments show that 2D ResNet34 and anisotropic 3D ResNet are the best 2D and 3D models, so we used these two CNNs as the basic model of the ensemble CNN. Different ensemble strategies will have an impact on the classification results, so we compared the diagnosis results of two ensemble strategies, including feature level ensemble strategy and decision-level ensemble strategy. In the feature level ensemble strategy (Feature-Ensemble), global average pooling was performed on the output feature maps of the last convolution of 2D and 3D CNNs, and then concatenated the pooled one-dimensional feature vectors. The fused feature vector was finally classified by the fully connected layer. In the decision-level ensemble strategy, 2D and 3D CNNs made decisions independently, and the final result was obtained by averaging the output of two CNNs.

**Fig. 7:**
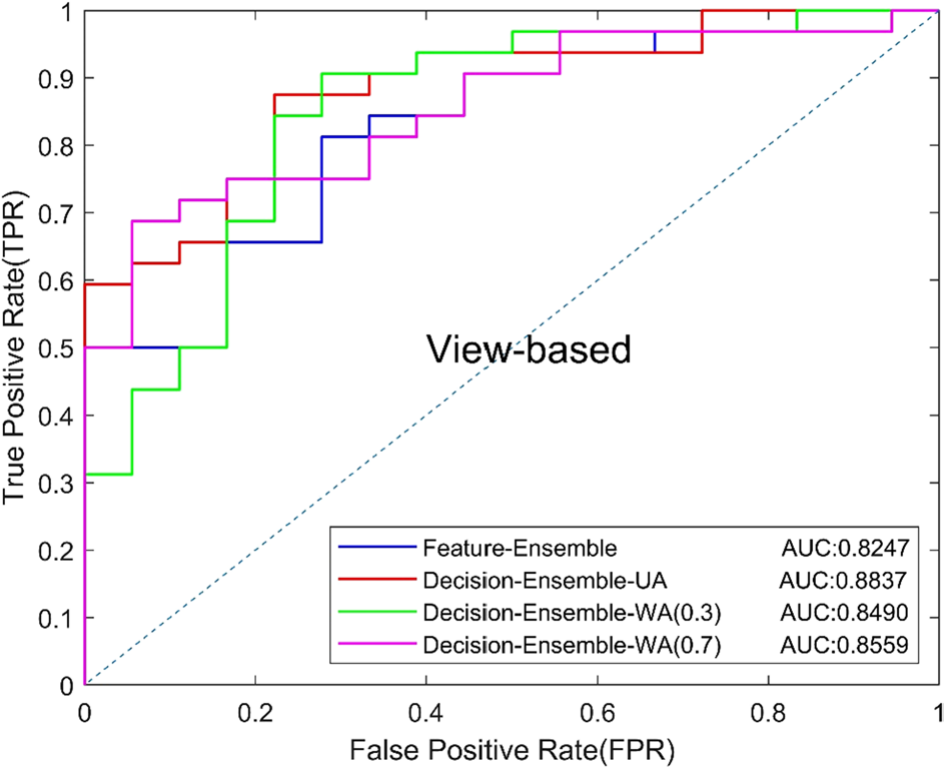
View-based ROC curves of different ensemble methods

**Fig. 8:**
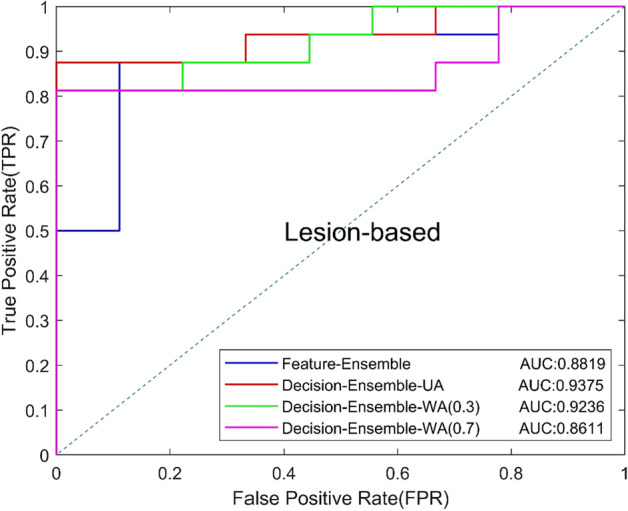
Lesion-based ROC curves of different ensemble methods

In addition, we compared the effects of unweighted average (Decision-Ensemble-UA) and weighted average (WA) on diagnosis results in the decision-level ensemble strategy, and explored the contribution of 2D ResNet34 and 3D anisotropic ResNet for the final results. The unweighted average means that the two CNNs have the same weight. For the WA, we set a weight of 0.3 [Decision-Ensemble-WA (0.3)] or 0.7 [Decision-Ensemble-WA (0.7)] for 2D ResNet34 to explore the influence of anisotropic 3D ResNet or 2D ResNet34 with a large weight on the results. Table 5 shows the experimental results, and the ROC curves based on view and lesion are shown in Figs. 7 and 8. The results show that, the decision-level ensemble method can improve the classification results of 2D ResNet34 and anisotropic 3D ResNet alone, the classification result of the features-level ensemble method is lower than 2D ResNet34 and anisotropic 3D ResNet alone, which indicate that feature level ensemble may bring feature redundancy and increases the difficulty of classification layer. In addition, decision-level ensemble with unweighted average is better than weighted average, which indicates that 2D focus slice features are equally important as 3D contextual features for MCs benign and malignant classification.

**Table 5:** Classification results of different ensemble methods

Models	AUC	ACC (%)	SEN (%)	SPEC (%)	Precision (%)	Recall (%)	*F*1 (%)
Feature-Ensemble	0.8247	76.00	87.50	55.56	77.78	87.50	82.35
Decision-Ensemble-UA	0.8837	82.00	84.38	77.78	87.10	84.38	85.71
Decision-Ensemble-WA(0.3)	0.8490	80.00	81.25	77.78	86.67	81.25	83.87
Decision-Ensemble-WA(0.7)	0.8559	74.00	75.00	72.22	82.76	75.00	78.69

### E. Comparison of radiomics and deep learning

**Fig. 9:**
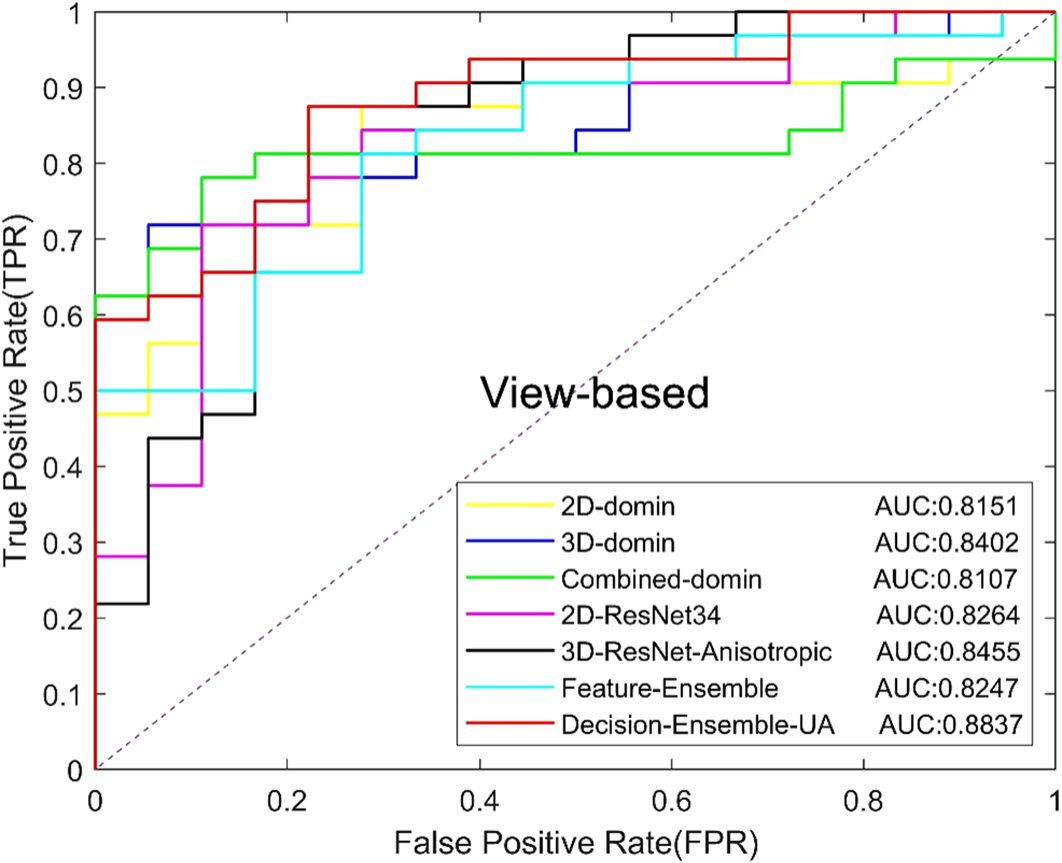
View-based ROC curves of radiomics method and deep learning method

**Fig. 10:**
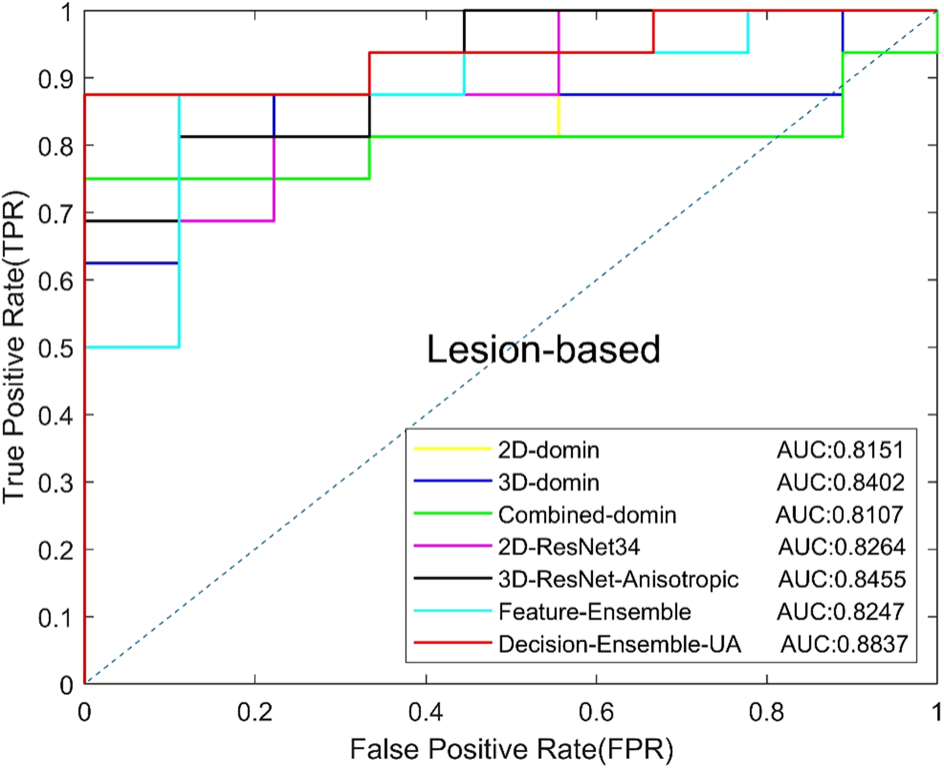
Lesion-based ROC curves of radiomics method and deep learning method

We compared the ensemble CNN with our the representative radiomics method [16]. In the radiomics method, we extracted 2D features from the focus slice and the maximum intensity projection image, and 3D features from volume. The Least Absolute Shrinkage and Selection Operator (LASSO) method was used for feature selection and the random forest was used for classification. We compared the performances of 2D features, 3D features and combined features in the classification of benign and malignant MCs. The experimental results are shown in Table 6, and the ROC curves based on views and lesions are shown in Figs. 9 and 10, respectively, where 2D-domain, 3D-domain and combined-domain represent the models using 2D, 3D and combined features in the radiomics method. The experimental results show that classification results of MCs using DL method are better than radiomics method. In addition, in the radiomics method or DL method, the results of features-level ensemble are lower than those of the 2D and 3D features alone.

**Table 6:** Classification results of radiomics method and deep learning

Methods	Models	AUC	ACC (%)	SEN (%)	SPEC (%)	Precision (%)	Recall (%)	*F*1 (%)
Radiomics [16]	2D-domain	0.8151	76.00	87.50	55.56	77.78	87.50	82.35
	3D-domain	0.8402	74.00	78.13	66.67	80.65	78.13	79.37
	Combined-domain	0.8107	72.00	81.25	55.56	76.47	81.25	78.79
The proposed method	2D-ResNet34	0.8264	76.00	78.13	72.22	83.33	78.13	80.65
	3D-ResNet-Anisotropic	0.8455	76.00	75.00	77.78	85.71	75.00	80.00
	Feature-Ensemble	0.8247	76.00	87.50	55.56	77.78	87.50	82.35
	Decision-Ensemble-UA	0.8837	82.00	84.38	77.78	87.10	84.38	85.71

### F. Comparisons with related works

**Fig. 11:**
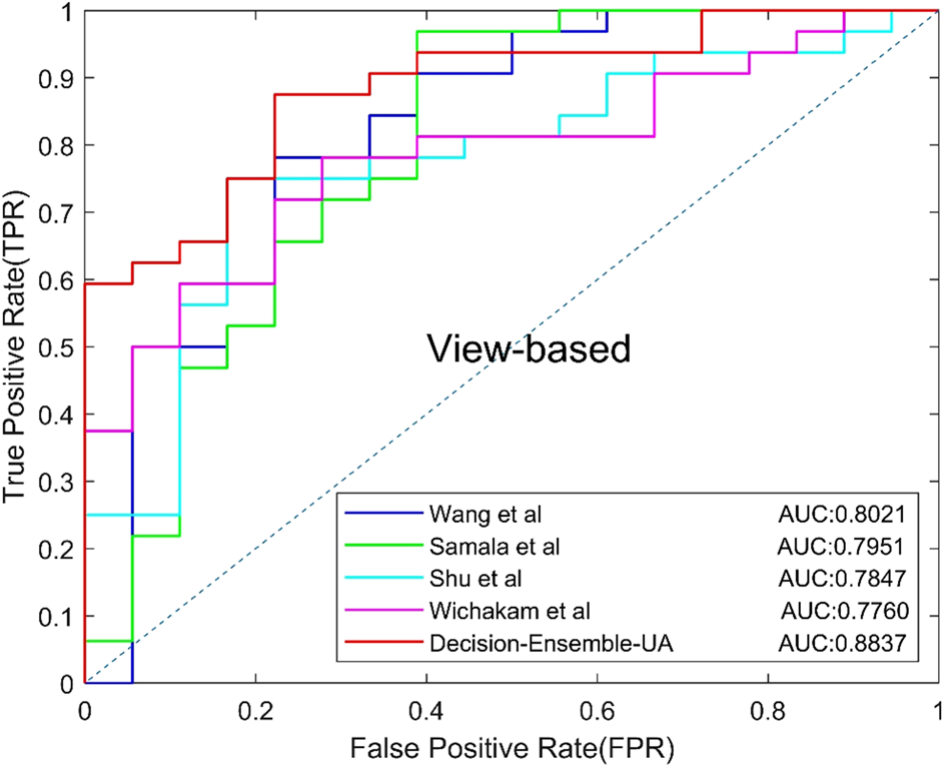
View-based ROC curves of related work

**Fig. 12:**
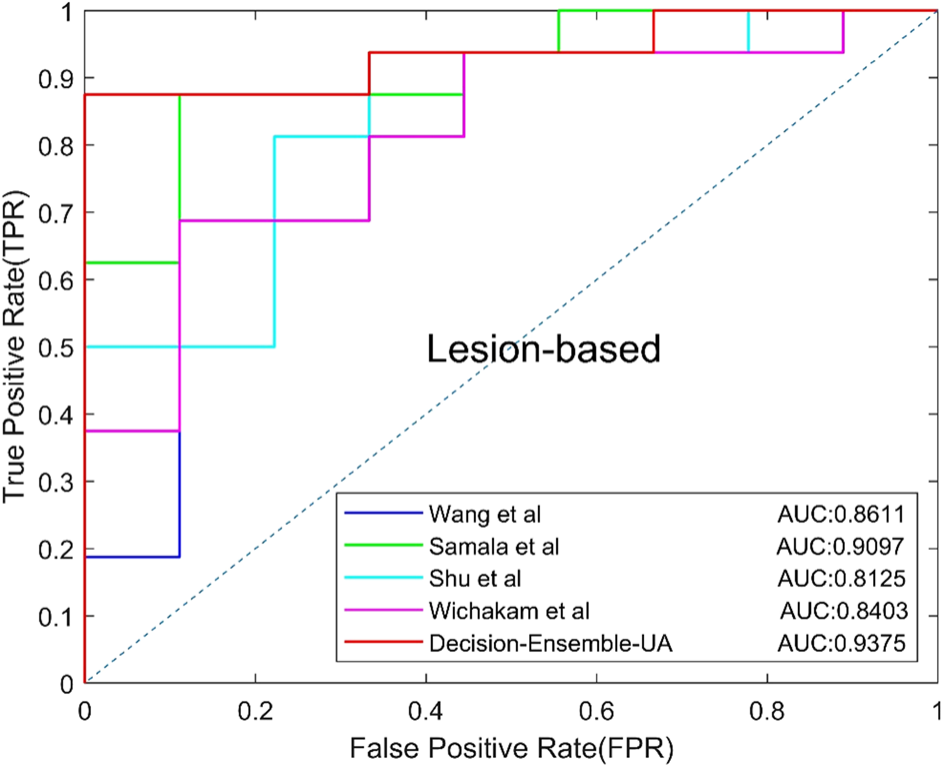
Lesion-based ROC curves of related work

So far, there are only a few CNN-based studies on classification of benign and malignant MCs focus on DBT, and the majority approaches are based on DM. So we reproduced four related works [18–22] on our dataset for comparison with the ensemble CNN, the parameters are recorded according the papers. The four related works include two DBT-based methods and two DM-based methods, the experimental results are shown in Table 7, and the ROC curves based on views and lesions are shown in Figs. 11 and 12, respectively.

**Table 7:** Classification results of related work

Methods	Models	AUC	ACC (%)	SEN (%)	SPEC (%)	Precision (%)	Recall (%)	*F*1 (%)
A	Samala et al. [19]	0.7951	72.00	71.88	72.22	82.14	71.88	76.67
B	Wang et al. [18]	0.8021	74.00	78.12	66.67	80.65	78.12	79.37
C	Wichakam et al. [21]	0.7847	72.00	78.12	61.11	78.12	78.12	78.12
D	Shu et al. [22]	0.7760	74.00	75.00	72.22	82.76	75.00	78.69
E	Decision-Ensemble-UA	0.8837	82.00	84.38	77.78	87.10	84.38	85.71

Method A and C are DBT-based methods. Samala et al. designed a CNN with 4 convolutional layers to classify MCs in MIP of DBT. Wichakam et al. used a 3D CNN with 8 convolutional layers to classify whether the input has lesions. Method B and D are DM-based methods, and the focus slice of DBT volume is used as the input. Wang et al. used a context-sensitive deep neural network to reduce false positives, in which two CNNs were used to extract the features of MCs in DM with size 95 × 95 and 9 × 9. Finally, the output features of two CNNs were integrated to obtain the final classification results. Shu et al. used DenseNet169 as the backbone network and added a new pooling structure for DM classification. Compared with the above four methods, the proposed method achieved the highest results on all metrics, indicating that the proposed method is more effective in classifying MCs.

## Discussion

In this study, we proposed an ensemble CNN to classify benign and malignant MCs in DBT. This model contained a 2D ResNet34 branch to extract focus slice features and an anisotropic 3D CNN branch to extract 3D contextual features, the final output of the model was the combination of 2D and 3D results. Compared with 2D CNN and 3D CNN alone, the ensemble CNN can achieve the best diagnostic results.

We first tested the validity of anisotropic 3D ResNet on the benign and malignant classification of DBT. Compared with the standard 3D ResNet, the AUC of anisotropic 3D ResNet was increased from 0.8299 to 0.84551, and specificity was increased from 61.11 to 77.78%. It indicated that, compared with the standard 3D convolution, the anisotropic 3D convolution can avoid the influence of DBT anisotropic resolution, and extract 3D contextual features effectively.

In the experiment of ensemble method, we experimented with the feature-level ensemble method and decision-level ensemble method, respectively. The AUC of the feature level ensemble method was 0.8247, which was worse than 2D ResNet34 (AUC 0.8264) and 3D ResNet (AUC 0.8455) alone. The experimental results show that feature level ensemble cannot improve model performance. The fusion of 2D features and 3D features may lead to feature redundancy and increased the difficulty of the classification layer, thus affect the performance of the classification model.

In the decision-level ensemble method, we used the unweighted average strategy and the weighted average strategy to integrate the 2D ResNet34 output and the anisotropic 3D ResNet output. In the weighted average strategy, we used the weights of 0.3:0.7 and 0.7:0.3, respectively, for the average. It can be seen that the AUC of unweighted average, weighted average by 0.3:0.7 and weighted average by 0.7:0.3 were 0.8837, 0.8490 and 0.8559, which were all higher than the AUC of 2D ResNet34 anisotropic 3D ResNet alone. The experimental results indicate that the ensemble method of decision-level ensemble can effectively improve the classified performance of benign and malignant MCs, and the unweighted average strategy achieved the best performance which indicates that the focus slice features and 3D spatial features have the same importance for classifying benign and malignant MCs in DBT.

We further compared our DL model with the radiomics method. The experimental results show that the proposed DL model achieved a better classification result than the previous radiomics method. Compared with the radiomics method, the AUC of the DL model is increased by 0.0435, and the *F*1 score is increased from 79.37 to 85.71%, indicating that the DL model can achieve more balanced results. During the radiomics method experiment, we found that the combination of 2D features and 3D features before the classifier did not improve the model performance, which is same as our experiment of feature level ensemble strategy.

Finally, we compared our method with four related works. Method A used a CNN with four convolutional layers to classify the MIP of DBT. Shallow CNN cannot extract more representative information, so the best classification result cannot be obtained. Method C used a standard 3D CNN to classify whether the DBT volume contained lesions, however, standard convolution cannot effectively extract 3D features of DBT with anisotropic resolution. Method B uses lesions with different sizes as input, but the sizes of MCs vary greatly, so it is difficult to choose the appropriate size and the performance may not be optimal. Method D added a new pooling structure to DenseNet169 to classify the MCs in the focus slice. However, 2D CNN does not make full use of 3D spatial information of DBT. The ensemble CNN can effectively utilize the 3D spatial information and 2D information of DBT, and use anisotropic convolution to avoid the influence of DBT anisotropic resolution. Compared with the four methods, the method proposed in this paper achieved the best results in AUC and *F*1 score.

## Conclusions

In this paper, a new ensemble CNN is proposed for the classification of MCs in DBT volume. The network has the following advantages. It is the first attempt to classify MCs in DBT with ensemble CNN. This CNN improves the diagnosis results by integrating the classification results of 2D ResNet34 and 3D ResNet. 3D ResNet is built by anisotropic 3D convolution, which can avoid the influence of DBT intra-slice and inter-slice anisotropic resolution on the results. We verify the effectiveness of the proposed method on a large clinically collected DBT dataset. In addition, compared with the radiomics method, the proposed deep learning can improve the classification results and effectively reduce the false positives. However, this method also has some limitations. The 3D bounding box of the MCs is manually delineated by the doctor, which is subjective. Therefore, the semi-supervised detection algorithm can be used to detect the boundary box of MCs.

## Methods

**Fig. 13:**
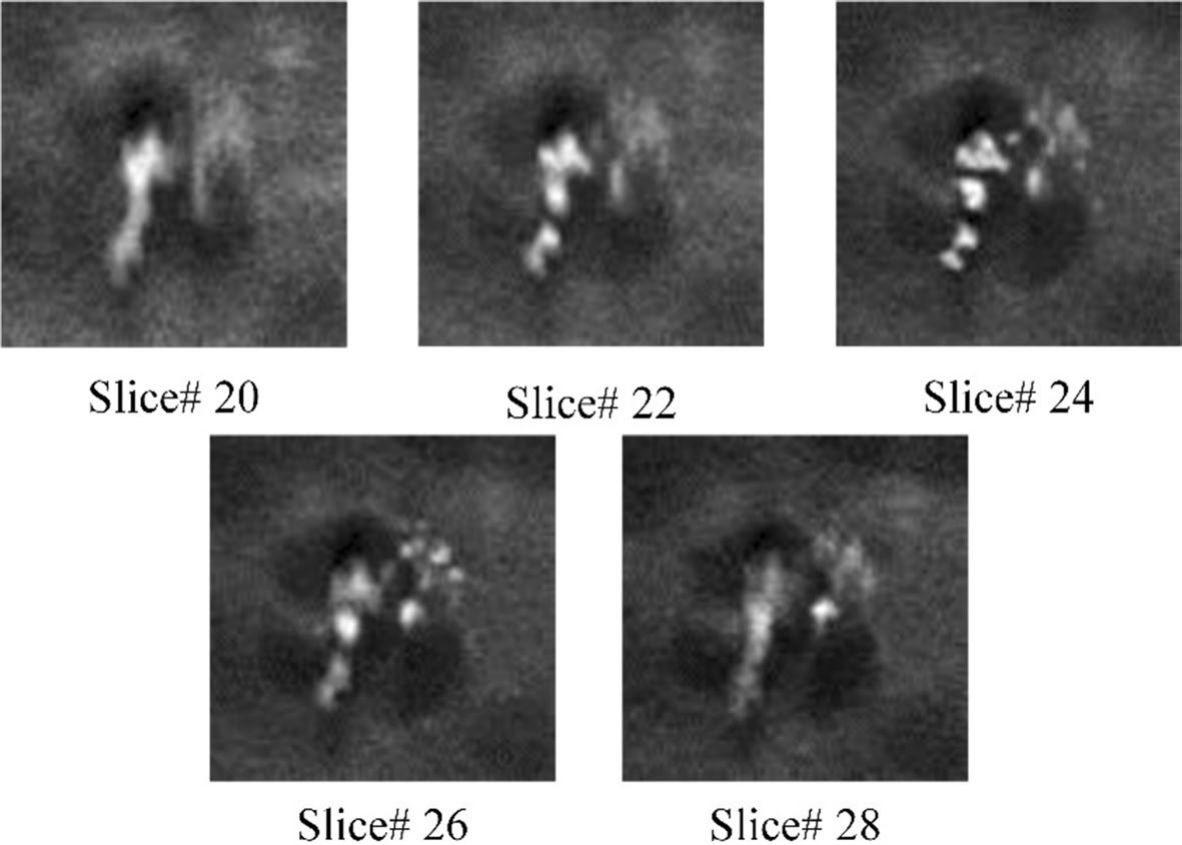
Morphology and distinctness of MCs in different slices. Slice#24 is the focus slice. Slice#22 and Slice#20 are the second and fourth slice on the left of the focus slice. Slice#26 and Slice#28 are the second and fourth slice on the right of the focus slice

**Fig. 14:**
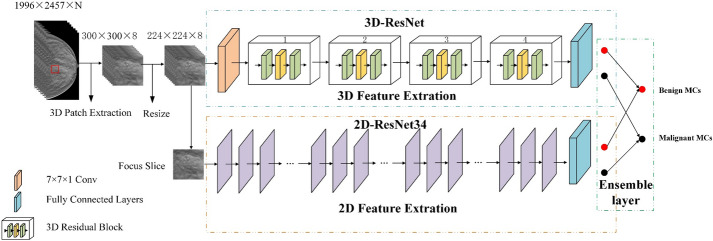
The overview of the ensemble CNN

DBT has the characteristic of anisotropic resolution. Its intra-slice resolution and inter-slice resolution are quite different. In addition, the MCs in non-focus slice are fuzzy, and the focus slice may contain more representative information. Figure 13 shows the morphologic appearances of different slices. It can be seen that the MCs is the clearest in the focus slice (Slice #24), and MCs will be fuzzy if the slice is farther away from the focus slice. In view of the above characteristics, an ensemble CNN was proposed to classify benign and malignant MCs in this paper, which integrated 2D ResNet34 and anisotropic 3D ResNet to classify benign and malignant MCs. Figure 14 shows the architecture of the proposed ensemble CNN, which consists of three parts: (1) 2D ResNet34 branch, aiming to extract intra-slice features of the focus slice, which has the clearest MCs appearance. (2) Anisotropic 3D ResNet branch, which uses anisotropic 3D convolution for spatial features extraction from DBT volumes with anisotropic resolution. (3) Decision-level ensemble layer, the classification results of 2D ResNet34 and anisotropic 3D ResNet were unweighted averaged to get the final classification results.

The proposed method was implemented by PyTorch framework, which is carried out an NVIDIA 2080Ti GPU. In the training stage, we set the initial learning rates of 2D ResNet34 and anisotropic 3D ResNet as 0.0001 and 0.00001, respectively. When the loss of the training set did not decrease, the learning rate decayed by a factor of 0.8. There are many efficient optimization algorithms [23–26]. In this work, we used Adam [26] with default settings as the optimizer. The fully connected layer was added to dropout (*p* = 0.5) to prevent overfitting [27].

### A. Data acquisition and preprocessing

With the approval of the Institutional Review Board, a dataset consisting of 462 DBT volumes from 236 patients was collected from the Department of Radiology, Nanjing Medical University Affiliated Hospital (Suzhou, China). The gold standard of benign and malignant in the dataset was determined by biopsy. There were 495 MCs in the 462 DBT volumes, of which 322 MCs were malignant and 173 MCs were benign. All DBT volumes were acquired by the Selenia Dimensions 3D Mammography system, and each breast was scanned twice two views (CC view and MLO view). All DBT volumes had intra-slice resolution of 100 μm and inter-slice resolution of 1 mm [9]. A radiologist with more than 5 years of DBT diagnosis experience used a 3D bounding box to mark MCs confirmed by biopsy. The bounding box was as close to surrounding the MCs as possible.

**Fig. 15:**
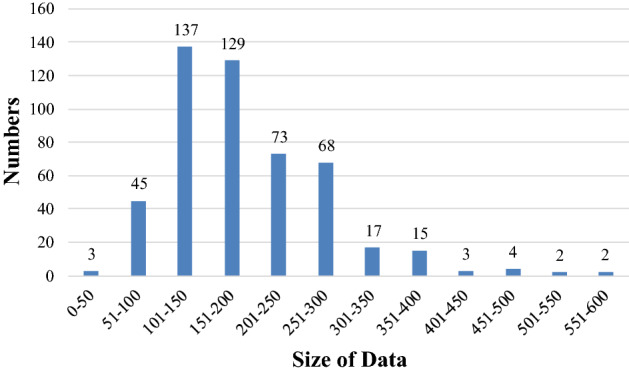
Statistical diagram of the size of the MCs area

We first analyzed the distribution of the size of MCs in slice, as shown in Fig. 15. It can be seen that the size of MCs in slice was mostly 300 × 300 and below, so we cut out a 300 × 300 × 12 volume from the center of the lesion, which can contain most of the MCs.

This experimental dataset included a total of 495 MCs, which were randomly divided into training, validation and test sets by 8:1:1. Considering the limitation of sample size, data augmentation [28] was used to increase the number of training samples, including translation, flip and rotation. In addition, different augmentation ratios were adopted for benign MCs and malignant MCs, respectively, to ensure the balance of training samples. The detailed information of the dataset is shown in Table 8.

**Table 8:** The characteristics of the dataset

Characteristics	Training set	Validation set	Test set
Benign patients	69	9	9
Malignant patients	116	17	16
Benign DBT volume	135	17	18
Malignant DBT volume	228	32	32
Benign MCs	138 (5106)	17	18
Malignant MCs	258 (5934)	32	32
Total MCs	396 (11,040)	49	50

The number in brackets in the training set is the number of augmented data

To accelerate the convergence of network training, it is necessary to normalize the data. Normalization does not change the image information and changes the pixel from 0–1023 to 0–1. In this paper, minimum–maximum normalization was used to perform linear transformation on the original data. The conversion formula is as follows:

7 $$P_{{{\text{out}}}} \left( {x,y} \right) = \frac{{P_{{{\text{in}}}} \left( {x,y} \right) - P_{\min } }}{{ P_{\max } - P_{\min } }},$$

where $P_{{{\text{in}}}} \left( {x,y} \right)$ is the pixel value at $\left( {x,y} \right)$ before conversion, and $P_{{{\text{out}}}} \left( {x,y} \right)$ is the pixel value at $\left( {x,y} \right)$ after conversion. $P_{{{\text{min}}}}$ is the smallest pixel value in the whole image, and $P_{{{\text{max}}}}$ is the largest pixel value in the whole image.

### B. Focus slice feature extraction

Generally, the MCs in DBT were distributed in multiple slices, which was clear in the focus slice, but fuzzy in the non-focus slice. The focus slice is the slice with the maximal gray level in DBT slices containing MCs. We used the method of Zhang et al. for the selection of focus slice. The calculation formula is as follows [15]:

8 $$\left\{ {\begin{array}{*{20}ll} {G\left( k \right) = \frac{{\sum\nolimits_{i = 1}^{M} {\sum\nolimits_{j = 1}^{{ N_{i} }} { p_{ij}^{k} } } }}{{\sum\nolimits_{i = 1}^{M} {N_{i} } }}} \\ { {\arg }_{k} \max G\left( k \right)} \\ \end{array} } \right.,$$

where $M$ is the number of individual microcalcifications in the MCs in the *k*th slice. $N_{i}$ is the number of pixels contained in the *i*th microcalcification, and $p_{ij}^{k}$ is the pixel value.

The focus slice may contain valuable representative features, so we used 2D ResNet34 to extract the intra-slice features of the focus slice. 2D ResNet34 was used for 2D focus slice features extraction, which can effectively solve the problem of gradient disappearance and gradient explosion caused by too deep CNN [29, 30]. Then we changed the number of nodes from 1000 to 2 to classify benign and malignant MCs.

### C. 3D anisotropic convolution

DBT data have an anisotropic resolution, while the standard 3D convolution kernel is isotropic, so it is difficult to fit intra-slice features and inter-slice features simultaneously [31]. Therefore, the anisotropic 3D convolution kernel was used to extract spatial features. The standard *k* × *k* × *k* convolution kernel can be divided into *k* × *k* × 1 intra-slice convolution and 1 × 1 × *k* inter-slice convolution.

Formula 3 is the standard isotropic 3D convolution:

9 $$F_{{{\text{out}}}} \left( {B,\; W_{{{\text{out}}}} ,\; H_{{{\text{out}}}} ,\; D_{{{\text{out}}}} ,\;C_{{{\text{out}}}} } \right) = F_{{{\text{in}}}} \left( {B,\; W_{{{\text{in}}}} ,\; H_{{{\text{in}}}} ,\; D_{{{\text{in}}}} ,\; C_{in} } \right) \cdot K_{k \times k \times k}^{{\left( { C_{{{\text{in}}}} , C_{{{\text{out}}}} } \right)}} ,$$

where $F_{{{\text{in}}}}$ and $F_{{{\text{out}}}}$ represent input feature map and output feature map. $W$, $H$ and $D$ represent width, height and thickness of feature map, respectively. *B* is the batch size. $K$ is the convolution kernel with *k* × *k* × *k* size, $C_{{{\text{in}}}}$ and $C_{{{\text{out}}}}$ are the channel dimension of the input feature map and output feature map, respectively.

The formula 4 is anisotropic 3D convolution:

10 $$F_{{{\text{out}}}} \left( {B,\; W_{{{\text{out}}}} ,\; H_{{{\text{out}}}} ,\; D_{{{\text{out}}}} ,\; C_{{{\text{out}}}} } \right) = F_{{{\text{in}}}} \left( {B,\; W_{{{\text{in}}}} ,\; H_{{{\text{in}}}} ,\; D_{{{\text{in}}}} ,\; C_{{{\text{in}}}} } \right) \cdot {K1}_{k \times k \times 1}^{{\left( { C_{{{\text{in}}}} , C_{{{\text{temp}}}} } \right)}} \cdot {K2}_{1 \times 1 \times k}^{{\left( { C_{{{\text{temp}}}} , C_{{{\text{out}}}} } \right)}} ,$$

where $K1$ and $K2$ are the intra-slice convolution kernel and inter-slice convolution kernel. $C_{{{\text{temp}}}}$ is the out channel dimension when extracting intra-slice features.

### D. 3D spatial features extraction

DBT is 3D data, which contains abundant 3D contextual information. Efficiently using 3D information of DBT can improve the classification performance of the model. In this study, we proposed a 3D ResNet with anisotropic convolution to extract 3D features from DBT.

**Fig. 16:**
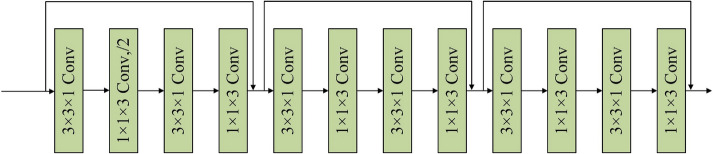
The architecture of the first residual block, represents the downsampling, and the size of the feature map is scaled to 1/2 of the original size

The anisotropic 3D ResNet was based on 3D ResNet34 architecture. Firstly, a 7 × 7 × 1 anisotropic convolution with 64 channels was adopted to extract inter-slice features slice by slice. Then 4 residual blocks were adopted to extract 3D contextual features. Thus, the information fusion between intra-slice and inter-slice was carried out in the feature maps level which avoided the influence of anisotropic resolution of the original DBT data. The numbers of output channels of the four residual blocks were 64, 128, 256, 512, respectively, and the standard 3D convolution kernels were replaced by anisotropic convolution kernels with kernel size 3 × 3 × 1 and 1 × 1 × 3. At the junction of two residual blocks, the channel number and size of the feature map are different. The Fig. 16 shows the first 3D residual block based on anisotropic convolution which contained three residual modules, each residual module contained four anisotropic convolution layers. The next three residual blocks were similar to the first one with 4, 6 and 3 residual modules.

### E. Ensemble strategy

3D anisotropic ResNet was used extracted 3D contextual features which can make full use of DBT data. However, 3D features may bring extra noise because of the fuzzy MCs in the slices which far away from the focus slices. The focus slice contained the clearest MCs, and 2D ResNet34 was used to extract focus slice features which can minimize the impact of noise. So the fusion of the 2D ResNet34 and anisotropic 3D ResNet results can improve classified accuracy and obtain a more stable and comprehensive model [32–36].

Decision-level ensemble strategy was used to integrate 2D Resnet34 and anisotropic 3D ResNet. In each epoch, 2D Resnet34 and anisotropic 3D ResNet were used to classify the MCs, respectively, and the final result was obtained by unweighted average of the two prediction probabilities.

## Data Availability

The data used and analyzed during the current study are available from the corresponding author on reasonable request.

## Abbreviations

MCs: Microcalcification clusters
CAD: Computer-aided diagnosis
DBT: Digital breast tomosynthesis
CNN: Convolution neural network
ROC: Receiver operating characteristic
AUC: Area under curve
DM: Digital mammography
RF: Random forest
MIP: Maximum intensity projection
DL: Deep learning
